# Molecular detection of feline leukemia virus in clinically ill cats in Klang Valley, Malaysia

**DOI:** 10.14202/vetworld.2021.405-409

**Published:** 2021-02-13

**Authors:** Kunambiga Mummoorthy, Abd Rahaman Yasmin, Siti Suri Arshad, Abdul Rahman Omar, Saulol Hamid Nur-Fazila, Prem Anand, Liew Wuan Hoong, Kiven Kumar

**Affiliations:** 1 Gasing Veterinary Hospital, 53 and 55, Jalan 5/58, Gasing Indah, 46000 Petaling Jaya, Selangor, Malaysia; 2 Department of Veterinary Laboratory Diagnosis, Faculty of Veterinary Medicine, Universiti Putra Malaysia, 43400 UPM Serdang, Selangor, Malaysia; 3 Laboratory of Vaccine and Biomolecules, Institute of Bioscience, Universiti Putra Malaysia, 43400 UPM Serdang, Selangor, Malaysia; 4 Department of Veterinary Pathology and Microbiology, Faculty of Veterinary Medicine, Universiti Putra Malaysia, 43400 UPM Serdang, Selangor, Malaysia

**Keywords:** feline leukemia virus, Malaysia, one-step reverse transcription polymerase chain reaction, phylogenetic analysis

## Abstract

**Background and Aim:**

Feline leukemia virus (FeLV) is classified as *Retroviridae gammaretrovirus*. FeLV occurs worldwide, including Malaysia. Thus far, only one decade-old study on molecular characterization of Malaysian FeLV isolates exists, which resulted in a scarcity of updated information of current FeLV isolates circulating in Malaysia. This study was conducted to determine the status of FeLV in clinically ill cats and to study the molecular characterization and phylogenetic relatedness of the current isolates.

**Materials and Methods:**

Convenience sampling was performed in 20 cats from the Gasing Veterinary Hospital in Selangor. Plasma and saliva samples were collected from 15 clinically ill cats and 5 healthy cats subjected to one-step reverse transcription-polymerase chain reaction with primers targeting a highly conserved gene of U3-LTR-gag.

**Results:**

Two clinically ill cats’ plasma and saliva samples tested positive for FeLV RNA. Partial nucleotide sequencing and phylogenetic analysis revealed that the current isolates were 94-99% homologous to the previous Malaysian and Japanese FeLV isolates.

**Conclusion:**

Current FeLV isolates from this study displayed higher similarity with the previous Malaysian isolates, signifying that a similar FeLV strain circulated among the cat population in Selangor.

## Introduction

Jarrett *et al*. first described the feline leukemia virus (FeLV) in 1964 [1]. Named *Retroviridae gammaretrovirus*, it is an enveloped, positive-sense, and single-stranded RNA virus. FeLV is a naturally occurring gammaretrovirus that infects domestic and ­sporadically feral cats [2-4]. At present, four FeLV subgroups of clinical importance exist, namely, FeLV-A, FeLV-B, FeLV-C, and FeLV-T. Subgroup FeLV-A is found in all naturally infected cats. Meanwhile, ­subgroup B arises from the recombination of endogenous FeLV with FeLV-A. In contrast, FeLV subgroups C and T are the mutated forms of FeLV-A [5]. Transmission of FeLV mostly occurs through direct contact with infected cats’ saliva and nasal secretions, predominantly during the sharing of food and water dishes, grooming, or aggressive behavior. Vertical transmissions may occasionally occur but are of little significance [6].

Remarkably, FeLV infection leads to various FeLV-related disorders such as the manifestation of immunosuppression, lymphoid or myeloid tumors, anemia, reproductive problems, immune complexes, enteritis, and other secondary disorders [5,6]. The infections are divided into four stages: The abortive infection (regressor cats), regressive infection ­(transient viremia followed by latent infection), progressive infection (persistent viremia), and focal or atypical infection [7]. The disease is highly contagious, occurring worldwide. The prevalence greatly varies with cats’ geographical distribution, health status, age, and population density [8]. Frequently, FeLV diagnosis is performed by detection of the p27 antigen using commercially available rapid test kits; reverse transcription-polymerase chain reaction (RT-PCR) assay testing is rare [9-11]. Conversely, the demonstration of the p27 antigen is relatively difficult during early viremia and latent infections. Conducted studies show that FeLV viral RNA and provirus DNA are better predictors of progressive and latent infections, respectively. Consequently, the severity and complexity of the disease could be reduced by obtaining appropriate samples and conducting a more sensitive diagnosis.

Previous studies highlight high prevalence rates of FeLV in both owned and free-roaming cat populations in Malaysia [8,12]. Nevertheless, only one study has been carried out on the molecular characterization of Malaysian FeLV isolates, performed approximately a decade ago.

As limited data exist on the current FeLV strain circulating in Malaysia, this study determines the status of FeLV in clinically ill cats and studies the molecular characterization and phylogenetic relatedness of the current isolates.

## Materials and Methods

### Ethical approval

Before proceeding with sample collection, approval from the Institutional Animal Care and Use Committee was obtained with the AUP No: /IACUC/AUP-FYP.2016/FPV. Informed consent was also given to cat owner’s prior sampling.

### Study location and period

A total of 20 animals were identified and sampled at the Gasing Veterinary Hospital, Bukit Gasing, Petaling Jaya, Selangor in December 2016. Of these, 15 cats were owned by clients and exhibited a wide variety of clinical signs such as respiratory signs (dyspnea, sneezing, and coughing), renal disease (dehydration, and halitosis), gastrointestinal signs (vomiting and/or diarrhea), depression, and anorexia. The remaining five cats were presented healthy during physical examination.

### Sampling

Convenience sampling was used in the present study. Clinically, ill cats were targeted, and samples were obtained only after owner consent allowed sampling. The clinically healthy cats were conveniently selected from resident hospital cats, which are usually kept for blood donation and mating. About 2 mL of blood was collected in EDTA tubes (either through cephalic, saphenous, or jugular vein), while oral swabs were collected using sterile swab sticks and placed immediately in a capped tube to stop it from drying out. Further related information regarding the cats was also obtained for each individual client in a structured form.

### RT-PCR analysis

Using innuPREP Virus DNA/RNA Kit (Analytik Jena, Germany), RNA from saliva and plasma samples were extracted according to the manufacturer’s instructions. Previously published primers used were U3F (5′-ACAGCAGAAGTTTCAAGGCC-3′) and G–R (5′-GACCAGTGATCAAGGGTGAG-3′), which targeted the U3-LTR-gag region [8]. Amplification of RNA from extracted plasma and saliva samples was carried out using one-step Access RT-PCR^®^ (Promega, USA) in a Mastercycler^®^ gradient thermal cycler (Eppendorf, Germany), with an expected amplicon size of 770 bp. The thermal cycling parameters for amplification of FeLV were set as follows: Reverse transcription for 20 min at 45°C and polymerase activation for 1 min at 95°C, followed by 40 cycles of 10 s denaturation at 95°C, 10 s annealing at 52°C, and 30 s extension at 72°C. The amplified PCR products were analyzed by gel electrophoresis using 1.5% (w/v) agarose gel (Vivantis, Malaysia).

### FeLV partial sequencing

RT-PCR product was subjected to DNA sequencing before it had been purified using the NucleoSpin Gel and PCR Clean-up kit (Macherey–Nagel, Germany) in ABI PRISM 3730xl Genetic Analyzer (Applied Biosystems, USA). The obtained sequences were analyzed for homology using the NCBI Basic Local Alignment Search Tool (BLAST) algorithm.

### Construction of phylogenetic tree

The phylogenetic tree was generated using Neighbor-Joining (NJ) with the 1000 bootstrap sampling method using MEGA-7. The Newick Export file was generated, and the tree was viewed and edited by the Interactive Tree of Life. The tree includes sequences from cat-2 UPM_FeLV_boboi-Malaysia (GenBank accession number: MW293981) and ­cat-14 UPM_FeLV_brownie-Malaysia (GenBank accession number: MW293982), as well as 23 other reference strains of FeLV, downloaded from GenBank^®^ (Supplementary Table-1) [12,13-19].

**Supplementary Table-1:** Reference isolates of FeLV downloaded from GenBank.

No.	Strain	Accession No.	Country	Source	References
1.	FeLV-UPM01	HQ197367	Malaysia	GenBank	[12]
2.	FeLV-UPM02	HQ197368	Malaysia	GenBank	[12]
3.	FeLV-UPM03	HQ197369	Malaysia	GenBank	[12]
4.	FeLV-UPM07	HQ197373	Malaysia	GenBank	[12]
5.	FeLV-UPM11	HQ197377	Malaysia	GenBank	[12]
6.	FeLV-UPM19	JF815542	Malaysia	GenBank	[12]
7.	FeLV-UPM20	JF815543	Malaysia	GenBank	[12]
8.	FeLV-UPM29	JF815552	Malaysia	GenBank	[12]
9.	FeLV Ricard	AF052723	USA	GenBank	[15]
10.	FeLV-FAIDS	M18247	USA	GenBank	[13]
11.	FeLV-GM1	D13922	UK	GenBank	[16]
12.	FeLV-TWK25	GQ465833	Taiwan	GenBank	Unpublished
13.	FeLV-TWK30	GQ327961	Taiwan	GenBank	Unpublished
14.	FeLV-Glasgow-1	KP728112	Scotland	GenBank	[17]
15.	FeLV-ON33	AB847229	Japan	GenBank	[14]
16.	FeLV-AT34	AB847164	Japan	GenBank	[14]
17.	FeLV-IT38	AB847183	Japan	GenBank	[14]
18.	914MGE	EU048352	Brazil	GenBank	[18]
19..	1286MG	EU090948	Brazil	GenBank	[18]
20.	843MG	DQ821500	Brazil	GenBank	[18]
21.	FeLV-MED68_C_CO_2018	MT229942.1	South America	GenBank	[19]
22.	FeLV_US_x1613_Fca2011	MF681664.1	USA	GenBank	Unpublished
23.	FeLV_US_x2655_Fca2018	MH116005.1	USA	GenBank	Unpublished
24.	UPM_FeLV_boboi-Malaysia	MW293981	Malaysia	This study	-
25.	UPM_FeLV_brownie-Malaysia	MW293982	Malaysia	This study	-

FeLV: Feline leukemia virus

## Results

**Figure-1:**
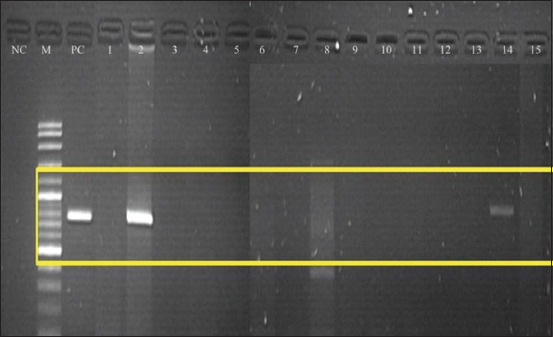
Reverse transcription-polymerase chain reaction assay of saliva of clinically ill cats using specific primer targeting U3LRT-gag region of Feline leukemia virus to produce 770 bp PCR products. Electrophoresis was carried out on 1.5 % (w/v) agarose gel. The target bands were conserved for all cats as shown in lane 2 (Cat-2) and lane 14 (Cat-14). Lane NC: Negative control, Lane PC 2: Positive control, Lane M: DNA marker 1kb.

**Figure-2:**
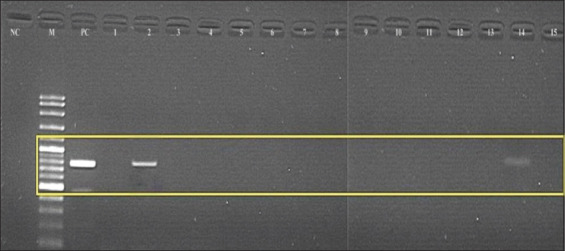
Reverse transcription-polymerase chain reaction assay of plasma of clinically ill cats using specific primer targeting U3LRT gag region of Feline Leukemia virus to produce 770 bp PCR products. Electrophoresis was carried out on 1.5% (w/v) agarose gel. The target bands were conserved for all cats, as shown in Lane 2 (cat 2) and Lane 14 (cat 14). Lane NC: Negative control. Lane PC: positive control Lanes 1 to 15 PCR product. The target bands were conserved for lanes 2 and 14. Lane M: DNA marker 1kb.

RT-PCR analysis revealed that none of the five healthy cats tested positive for FeLV RNA. Meanwhile, of the 15 clinically ill cats, two were positive for FeLV RNA in both plasma and saliva samples. Figures-1 and 2 show the gel electrophoresis of the positive DNA of the cats aligned with the positive control at the band (770 bp). Both cats were male; one of them was castrated (a stray free roamer), while the other cat was uncastrated and from a multi-cat household.

**Figure-3:**
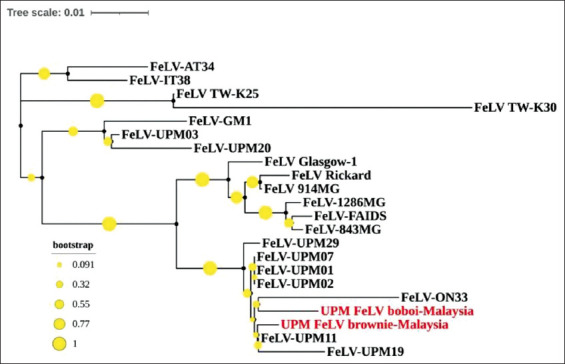
Neighbor-joining phylogenetic tree of feline leukemia virus (FeLV) isolates. Bootstrap values were obtained from 1000 bootstrap replicates. The scale bar corresponds to 0.01 changes per nucleotide. Current isolates are denoted as UPM FeLV-Boboi-Malaysia and UPM FeLV-Brownie-Malaysia (highlighted in red) is share same branches with the previous Malaysian isolates (UPM isolates) and only FeLV-UPM03 and FeLV-UPM20 are formed sister-branches with FeLV-GM1 A neighbor-joining phylogenetic tree was constructed based on the U3LTR-gag sequences using MEGA7 software. The tree reliability was assessed using 1000 bootstrap replicates.

BLAST analysis revealed that the partial sequence from the current isolates was more than 90% identical to FeLV reference sequences obtained from the GenBank^®^ (National Center for Biotechnology Information, USA). The comparison revealed that the partial U3LTR-gag regions of the current isolates were 94-99% homologous to the previous local isolates. Phylogenetic analysis using NJ with the 1000 bootstrap sampling method using MEGA-7 demonstrated that current isolates denoted as UPM FeLV-Boboi-Malaysia (MW293981) and UPM FeLV-Brownie-Malaysia (MW293982) share equal branches with the previous Malaysian isolates (UPM isolates). Next, only FeLV-UPM03 and FeLV-UPM20 formed sister branches with FeLV-GM1 (Figure-3).

## Discussion

This study primarily targeted clinically ill cats by taking plasma and saliva samples for the detection of FeLV as well as characterizing the positive isolates molecularly. Early FeLV diagnosis is crucially important as the manifestation of the infection is generally nonspecific. By conducting the study using plasma and saliva samples, the indication of the stage of FeLV infection was facilitated. Detection of FeLV nucleic acid in plasma indicated that the cat might be in the early stage of infection (regressive infection) or chronic stage of infection (progressive infection) as the presence indicates signs of viremia [5,10]. To distinguish regressive and progressive infections, peripheral blood should be tested at least 16 weeks after the first antigen testing, as regressively infected cats will turn negative at the latest 16 weeks after infection, while progressively infected cats will remain positive [10]. Meanwhile, the detection of FeLV viral nucleic acid in the saliva might indicate the cats are in the progressive stage of infection due to the shedding of the virus in the saliva [6,10]. In this study, FeLV RNA was detected in both the saliva and plasma samples from two cats, which likely indicates both cats were in the progressive stage of infection (also known as persistent viremia). These cats prospectively could develop FeLV-related disorders and be infectious to other cats from active shedding of the virus.

Risk factors associated with the development of FeLV in hosts include cats from multi-cat households and those that are semi- or free roamers [8]. Furthermore, the risk is higher for sick cats to be infected by FeLV as compared to healthy cats [2,8]. Other risk factors may include the neutered status of the cats in which intact males are more prone to FeLV infection as compared to castrated males [3,8]. In this study, one of the cats was a male, castrated, and stray (free-roaming cat), while the other cat was an uncastrated male from a multi-cat household. Both the cats were clinically ill and seemed to fit the increased risk factor of FeLV infection [8].

FeLV, true to its nature as a retrovirus, is subjected to genetic variation due to error-prone replication and recombination processes [10,20]. In this study, the U3LTR-gag region of naturally occurring exogenous FeLV was partially sequenced and subjected to phylogenetic analysis. The sequence alignment revealed a 94-99% similarity to the previous Malaysian isolates. A high-sequence homology was observed among the FeLV isolates derived from different geographic and temporal clusters, as reported by Bande *et al*. [12]. Donahue *et al*. [13] also reported findings of strong sequence conservation (>97%) among their FeLV isolates, due to the conserved nature of the U3 segment of the LTR and gag regions. In this study, the FeLV isolates were located within the same cluster in the phylogenetic tree as most of the previous Malaysian FeLV isolates. This could be due to their similar ancestral lineage.

## Conclusion

Based on the partial sequence of the U3LTR-gag region, this study demonstrated that the current Malaysian FeLV isolates are highly homologous with the previous Malaysian and Japan FeLV isolates. This suggests a possible circulation of a similar FeLV strain among Malaysian local cats. Information about the current FeLV genotypes that are circulating in Malaysia requires further investigations of FeLV envelope protein and whole-genome analysis.

## Authors’ Contribution

KM, ARY, and KK: Writing-original draft preparation, methodology and software. ARY, SSA, ARO, and SHN: Supervision and conceptualization. LWH: Methodology and software. PA: Sampling and methodology. All authors have read and agreed to the published version of the manuscript.
